# A dosimetric comparison of different radiotherapy modalities for Non-Resected oligometastatic liver Disease^☆^

**DOI:** 10.1016/j.ctro.2025.100947

**Published:** 2025-03-13

**Authors:** Cas Stefaan Dejonckheere, Mateusz Bilski, Younèss Nour, Davide Scafa, Paweł Cisek, Katarzyna Korab, Julia Ponikowska, Ewa Wojtyna, Sylwia Sroka, Fabian Kugel, Molina Grimmer, Jasmin Holz, Stephan Garbe, Patrick Eich, Eleni Gkika, Gustavo Renato Sarria, Julian Philipp Layer

**Affiliations:** a Department of Radiation Oncology, University Hospital Bonn 53127 Bonn, Germany; b Department of Radiotherapy, Medical University of Lublin 20059 Lublin, Poland; c Department of Brachytherapy, St. John’s Cancer Center, 20090 Lublin, Poland; d Department of Radiotherapy, St. John’s Cancer Center, 20090 Lublin, Poland; e Department of Medical Physics, St. John’s Cancer Center, 20090 Lublin, Poland; f Institute of Experimental Oncology, University Hospital Bonn 53127 Bonn, Germany

**Keywords:** Liver metastasis, Oligometastasis, Interventional radiotherapy, HDR brachytherapy, Stereotactic body radiation therapy, Electronic brachytherapy, Dosimetry

## Abstract

**Purpose:**

The role of radiotherapy in the context of limited liver metastases is emerging rapidly with a variety of available technical approaches. Here, we provide a dosimetric comparison of three different radiotherapy modalities for non-resected liver metastases.

**Methods:**

This multicentric study included 30 patients previously treated for solitary liver metastases. Interstitial high dose-rate brachytherapy (HDR), stereotactic body radiation therapy (SBRT), and kilovoltage electronic brachytherapy (eBT) with a single dwelling point, as an in this context novel modality, were planned with a 25-Gy single fraction and dosimetrically compared. The primary endpoint was difference in healthy liver tissue exposure. Secondary endpoints included target volume coverage, dose to the surrounding organs at risk (OARs), and radiation treatment time.

**Results:**

The median (range) lesion volume was 9.3 (2.5 − 29.7) cm^3^. Electronic BT yielded a significantly better OAR dose sparing compared to SBRT. Radiation exposure to the healthy liver was significantly lower with eBT (V9.1 Gy 13.8 [3.4–41.6] cm^3^) and HDR (49.2 [12.7–116.8] cm^3^) when compared with SBRT (98.8 [54.3–303.7] cm^3^; *p* < 0.001 for both). Mean treatment time was shortest for SBRT (6.1 min vs. 7.8 min for HDR [*p* = 0.003] vs. 16.1 min for eBT [*p* < 0.001]). Equally, CTV D90% and D95% were superior for SBRT (*p* < 0.001 vs. HDR and eBT).

**Conclusion:**

Minimally invasive single-catheter eBT provides a new potential approach in the context of unresectable liver metastases, with optimal OAR dose sparing, yet suboptimal target volume coverage. Combining the advantages of both modalities, a multi-dwelling eBT approach as in HDR might ease applicability in clinical routine with optimized target coverage while maintaining excellent OAR doses. Future prospective investigations are required to further define its role within well-established liver-directed therapies.

## Highlights

• Dosimetric comparison including SBRT, HDR, and electronic BT for liver metastases.
• Shorter treatment times and superior target coverage with established SBRT and HDR.
• Lowest OAR exposure with HDR and eBT, including healthy liver.
• Further investigation of eBT needed to reduce treatment time and improve target coverage.

## Introduction

Liver metastases develop in up to 5 % of cancer patients and generally carry a poor prognosis, with a 1-year survival rate of approximately 15 % [1]. Over the past decades, metastasis-directed treatment has improved substantially, with different locoregional modalities becoming more widely available, including transarterial chemoembolization (TACE), high-intensity focused ultrasound (HIFU), radiofrequency ablation (RFA), microwave ablation (MWA), or transarterial radioembolization (TARE; also known as selective internal radiotherapy or SIRT) [2,3]. The more recent introduction of targeted therapies for metastatic patients has also introduced durable local control in some instances [2]. Radiotherapy was long thought unsuitable in the context of liver metastases, but recent advances in radiation technique (e.g. image-guidance, real-time adaptive planning) and set-up (e.g. surface scanning, respiratory gating) have led to the introduction of stereotactic body radiation therapy (SBRT) as a definitive treatment modality, with recent findings highlighting significant antineoplastic activity and excellent local control rates of about 80 − 100 % [4]. The use of multicatheter interstitial brachytherapy (BT) with a 192-Ir high dose-rate (HDR) afterloader has been demonstrated as a safe and effective treatment modality for non-resectable liver malignancies, with a superior dose coverage of the target volume while reducing the radiation-exposed liver volume [[5], [6], [7], [8], [9], [10]]. Dosimetric comparisons with SBRT highlight its superiority in both areas [11,12].

As randomized trials between different modalities are scarce, the favored strategy remains unclear, but the mainstay in suitable candidates (especially in the oligometastatic setting) remains surgery [3,13,14]. In the past, anatomic resections were performed (i.e. the removal of entire liver segments) with subsequent functional impairments, potentially making patients unfit for required (future) systemic therapies [15]. Improvements in surgical technique and a better understanding of tumor spread and biology have led to more tissue-sparing approaches, leading to a reduced surgery-related morbidity and mortality, while largely maintaining oncological outcomes [16,17]. Non-anatomic resections, however, pose an increased risk of local recurrence, which could potentially lead to higher morbidity or mortality[18].

In order to resolve the surgical conundrum, we previously proposed the use of kilovoltage intraoperative radiotherapy (IORT) or electronic BT (eBT) to the resection cavity as a novel adjuvant treatment modality for resectable liver metastases, which could potentially improve local control [19]. In a dosimetric comparison with single-fraction stereotactic radiosurgery (SRS) to the resection cavity, a significant reduction in healthy liver radiation exposure was observed, which could potentially improve postoperative prognosis and keep patients fit for further lines of treatment [19]. Here, we aim to test whether these advantages also translate to the definitive setting, potentially providing a minimally-invasive and easily applicable alternative to SBRT and HDR. We report a dosimetric comparison of all three available radiotherapy modalities (HDR, SBRT, eBT) for non-resected liver metastases to compare established and potentially novel therapeutic options.

## Materials and Methods

### Patient selection and planning procedure

**Fig. 1:**
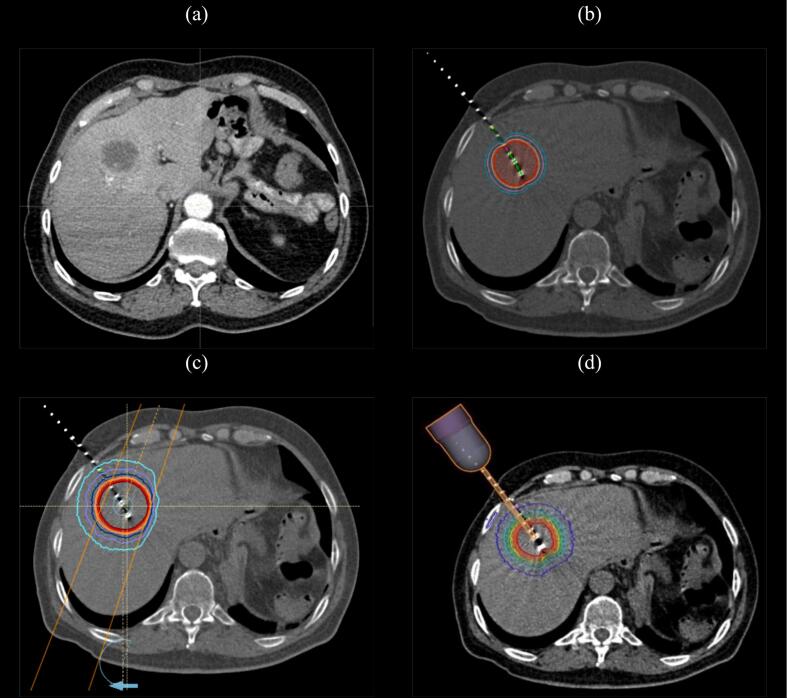
Exemplary case with a solitary non-resected liver metastasis in segment VIII **(a)** depicting the dose distribution profile of interstitial high dose-rate brachytherapy **(b)**, stereotactic body radiation therapy **(c)**, and electronic brachytherapy **(d)**. A single 25-Gy fraction was prescribed for all treatment modalities.

Data from patients with solitary, non-resected liver metastases (longest diameter < 4 cm) from any primary tumor with an uninvolved liver volume > 700 cm^3^ were retrospectively screened and 30 patients were randomly selected from an institutional database. All patients had received HDR between 2017 and 2023. A contrast-enhanced simulation computer tomography (CT) scan in supine position with both arms raised (3-mm slice thickness) was used to plan all treatment modalities (SOMATOM, Siemens Healthineers, Munich, Germany). Organs at risk (OARs) were delineated following international guidelines and included the healthy (i.e. uninvolved) liver, biliary tract, duodenum, ipsilateral kidney, spinal cord, stomach, heart, ribs, great vessels, gallbladder, small/large bowel, and esophagus. Dose constraints were adopted from a phase 1 dose-escalation trial of single-fraction SRS for liver metastases [20]. Biliary tract and ipsilateral kidney tolerance were defined according to related publications [21,22]. All OAR constraints are summarized in Table 1. To ease a direct comparison, an equivalent dose of 25 Gy in a single fraction, prescribed on the 100 % isodose, was chosen for all three treatment scenarios. Prespecified dosimetric parameters and irradiation times were assessed for all three modalities separately (Fig. 1).

**Table 1:** Summary of constraints for organs at risk (OARs), which were the same for all three treatment scenarios. D_max_ was defined as dose received in ≤ 0.035 cm^3^. GTV = gross tumor volume; Gy = Gray.

**OAR**	**Constraints**
Healthy liver (liver – GTV)	700 cm^3^ < 9.1 Gy; D_66%_ < 10 Gy
Biliary tract	D_max_ < 25 Gy
Duodenum	5 cm^3^ < 11.2 Gy
Ipsilateral kidney	10 Gy < 33 %
Spinal cord	D_max_ < 14 Gy; 1.2 cm^3^ < 7 Gy
Stomach	D_max_ < 12.4 Gy; 10 cm^3^ < 11.2 Gy
Heart	D_max_ < 22 Gy
Rib	D_max_ < 30 Gy; 1 cm^3^ < 23 Gy
Great vessels	1 cm^3^ < 27 Gy
Gallbladder	D_max_ < 20 Gy
Bowel	D_max_ < 15.4 Gy
Esophagus	D_max_ < 24 Gy; 1 cm^3^ < 15 Gy

#### Interstitial high dose-rate brachytherapy

With the patient under general anesthesia, an experienced radiation oncologist inserted flexible needles of 200- or 320-mm length into the lesion under CT-guidance. Depending on volume and location of the liver metastasis, 1 to 3 needles were used. After insertion, a new CT scan was performed and fused with the contrast-enhanced CT to define the clinical target volume (CTV). Treatment plans were calculated using HDR BRAVOS or GammaMedplus iX (Varian Medical Systems, Palo Alto, CA, USA), using a 192-Ir isotope. Afterwards, they were optimized using the VEGO TG-43 vol Optimization computational algorithm (Varian Medical Systems, Palo Alto, CA, USA). Treatment time was defined as crude radiation time, i.e. without preparation or insertion of needles or treatment planning, as this is highly variable and depends on the number of needles, location of the lesion, and experience of the treating team. As treatment time is mainly dependent upon the current activity of the radioactive source, a nominal source value of 10 Ci was used in all cases. Therefore, we were able to compare plans implemented over several years.

#### Stereotactic body radiation therapy

The target volume was defined on the contrast-enhanced simulation CT. The gross tumor volume (GTV) was defined, with an in this context 5-mm margin to create the planning target volume (PTV), allowing for a direct comparison of the three treatment modalities. Treatment plans were simulated for a Versa HD linear accelerator (Elekta, Stockholm, Sweden) equipped with the Agility multileaf collimator. The density of the applicators and resulting artefacts were overwritten with water density, to simulate normal liver tissue. The Active Breathing Coordinator (Elekta, Stockholm, Sweden) feature was used. Planning was performed in RayStation (RaySearch Laboratories, Stockholm, Sweden) with a limited arc range (180 to 200 degrees, with a maximum of 240 degrees) using the volumetric-modulated arc therapy (VMAT) technique (2 arcs) and a 6 MV photon energy. The Monte Carlo calculation algorithm was used with an uncertainty of 0.5 %. Treatment time was defined as beam-on time to deliver the prescribed dose (i.e. not including patient positioning and matching).

#### Electronic brachytherapy

To build a homogeneous collective of eBT treatment plans, a single rigid-needle applicator was used, following the trajectory of the main HDR catheter and respecting anatomic boundaries. The target volume was the same one as defined for HDR. Electronic BT calculations were performed using Radiance (GMV SA, Madrid, Spain), employing a Monte Carlo simulation for low-energy X-rays to be delivered with the INTRABEAM 600 (Carl Zeiss Meditec, Oberkochen, Germany). The assessment of treatment time for eBT was identical to HDR.

### Endpoints

The primary endpoint was the difference between modalities in radiation exposure to the healthy liver tissue, with the goal constraint set at D_700ccm_ < 9.1 Gy. Secondary endpoints were target volume coverage, including various parameters, and dose to the surrounding OARs, as well as radiation treatment time.

### 3 Statistical analysis

Mean with standard deviation (SD) and median with range metrics were calculated and reported for all applicable data. Direct comparisons were performed across the different treatment modalities for the different variables. Statistical differences were assessed with the Wilcoxon signed-rank test for continuous variables, assuming *p* ≤ 0.05 as the level of significance. Data were managed using Microsoft Excel version 16 (Microsoft, Redmond, WA, USA), SPSS Statistics version 27 (IBM, Armonk, NY, USA), GraphPad Prism version 10 (GraphPad Software, San Diego, CA, USA), and *R* version 4.2.2 (*R* Core Team 2022). Graphical elements were created using GraphPad Prism, *R* with the ggplot2 package and Adobe Illustrator 2023 (Adobe Inc., San José, CA, USA).

### 4 Ethics statement

This study was conducted in accordance with the principles of the Declaration of Helsinki. This trial was approved by the Institutional Review Board of the University Hospital Bonn, Germany on January 4, 2024 (449/23-EP).

## Results

### 1 Patient and lesion Characteristics

Thirty patients with a solitary non-resected liver metastasis were included. The median (range) lesion diameter was 26.7 (15.2 − 38.2) mm, resulting in a median lesion volume (GTV) of 9.3 (2.5 − 29.7) cm^3^. The uninvolved liver volume was 1450.2 (1085.8 − 2227.2) cm^3^. As for target structures, the defined GTVs for HDR and eBT were 9.3 (2.5 − 29.7) cm^3^, significantly smaller than the SBRT PTVs with 25.6 (11.4 − 59.2) cm^3^ (*p* < 0.001). HDR used a median number (range) of 1 (1 − 3) needles. All eBT plans used a single applicator.

### 2 Treatment time

**Fig. 2:**
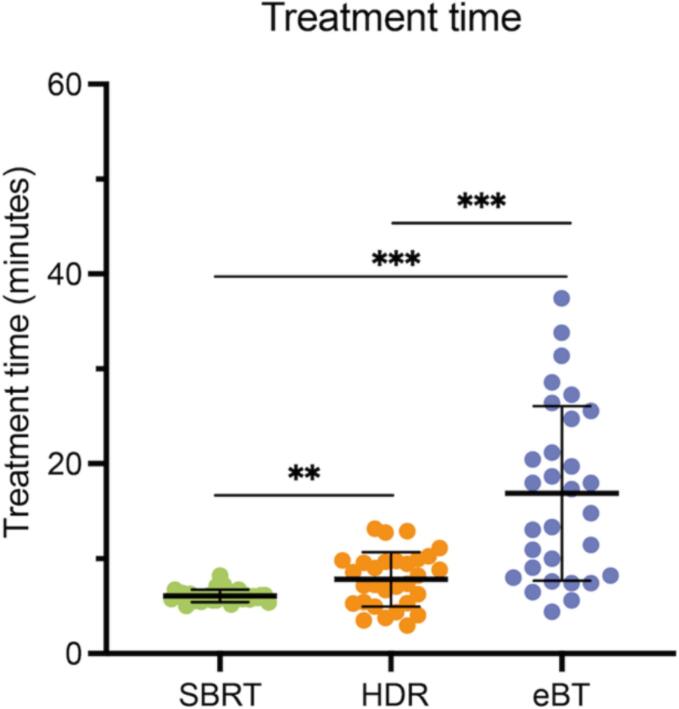
Differences in treatment time across all three radiotherapy modalities. Dot plots showing individual time values in minutes for stereotactic body radiation therapy (SBRT, green), high dose-rate brachytherapy (HDR, orange), and electronic brachytherapy (eBT, blue) of respective patients (*n* = 30). Lines indicate mean with standard deviation. Wilcoxon signed-rank test; ** *p* < 0.01; *** *p* < 0.001. (For interpretation of the references to color in this figure legend, the reader is referred to the web version of this article.)

No treatment time exceeded 40 min. With a median delivery time of 6.1 min, SBRT was significantly faster than both HDR (7.8 min; *p* = 0.003) and eBT (16.1 min; *p* < 0.001), as summarized in Fig. 2.

### 3 Target volume coverage

**Fig. 3:**
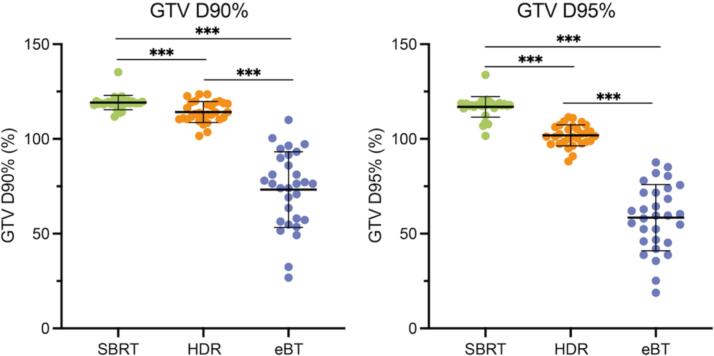
Differences in target volume coverage between all three radiotherapy modalities. Dot plots showing individual CTV D90% and D95% coverage (in %) for stereotactic body radiation therapy (SBRT, green), high dose-rate brachytherapy (HDR, orange), and electronic brachytherapy (eBT, blue) of respective patients (*n* = 30). Lines indicate mean with standard deviation. Wilcoxon signed-rank test; *** *p* < 0.001. GTV = gross tumour volume. (For interpretation of the references to color in this figure legend, the reader is referred to the web version of this article.)

SBRT provided the highest target volume coverage with a median CTV D90% and D95% of 118.9 % and 117.8 %, respectively. This was significantly superior to 113.5 % and 101.4 % for HDR (*p* < 0.001) and to 76.4 % and 59.6 % for eBT (*p* < 0.001). Results are summarized in Fig. 3.

### 4 Organ at risk doses

**Fig. 4:**
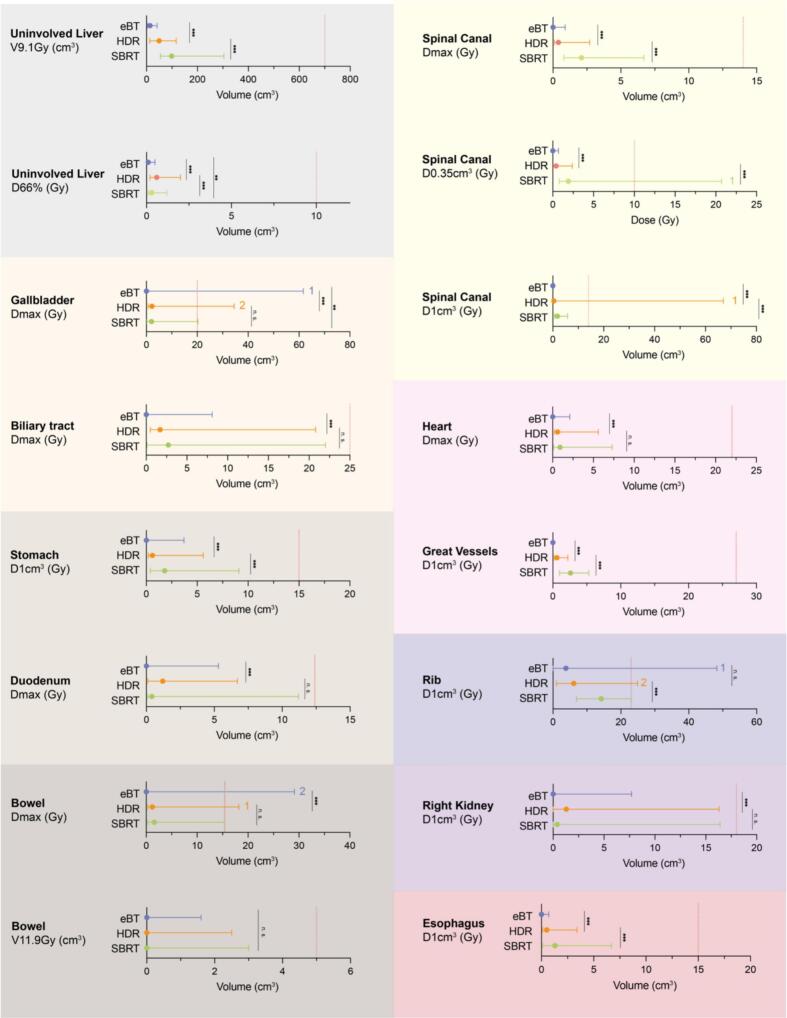
Comparison of dose distribution in selected organs at risk for all three radiotherapy modalities. Dots indicate median, whiskers minimum and maximum. Colors refer to electronic brachytherapy (eBT, blue), high dose-rate brachytherapy (HDR, orange), and stereotactic body radiation therapy (SBRT, green). Red dotted lines indicate dose constraints as specified under *Methods* and numbers beside lines indicate the number of patients exceeding the dose constraint mentioned. Wilcoxon signed-rank test; n. s. = not significant; ** *p* < 0.01; *** *p* < 0.001. Gy = Gray. (For interpretation of the references to color in this figure legend, the reader is referred to the web version of this article.)

**Fig. 5:**
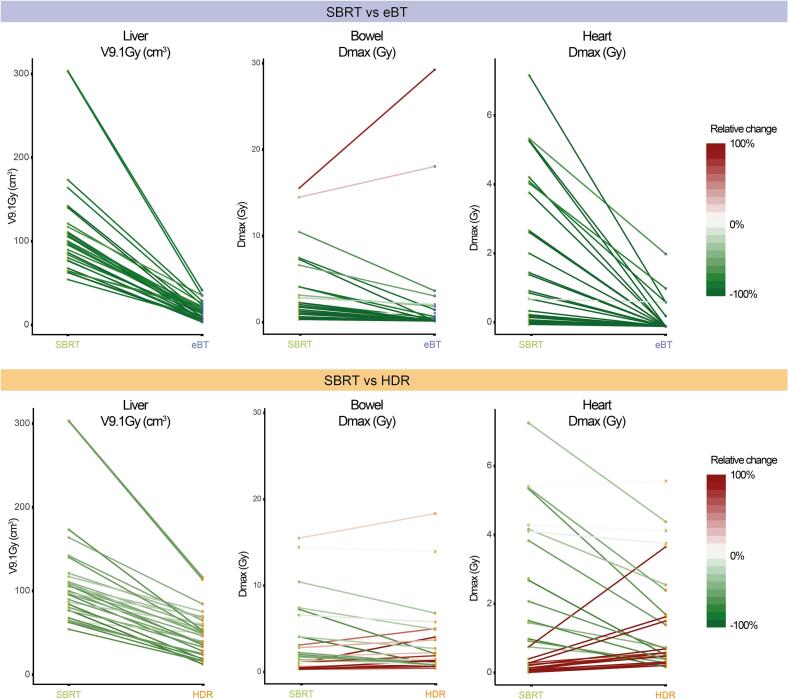
Relative changes of dose distribution for selected organs at risk. Colored dots indicate individual dose values (*n* = 30) for stereotactic body radiation therapy (SBRT, green) and electronic brachytherapy (eBT, blue) in the upper panel and for SBRT (green) and high dose-rate brachytherapy (HDR, orange) in the lower panel. Connecting lines indicate relative change (in %) as depicted in the color-labeled legend. Gy = Gray. (For interpretation of the references to color in this figure legend, the reader is referred to the web version of this article.)

With few exceptions, most of the constraints were met in all modalities. However, doses in nearly all OARs were significantly lower for HDR and eBT plans (Fig. 4). Regarding the primary endpoint, radiation exposure to the healthy liver tissue, median (range) V9.1 Gy was 13.8 (3.4–41.6) cm^3^ for eBT versus 49.2 (12.7–116.8) cm^3^ for HDR and 98.8 (54.3–303.7) cm^3^ for SBRT (*p* < 0.001 for both). Particularly eBT yielded a significantly better OAR dose sparing compared to SBRT, resulting in relative OAR dose reductions for the majority of patients (Fig. 5). Full dosimetric data are provided in Supplementary Table 1.

## Discussion

The therapeutic armamentarium for patients with liver metastases is expanding rapidly. The main goal in patients with limited liver involvement is yielding optimal local control, thus preventing further spread, while mitigating damage to the healthy liver, keeping patients fit for sequential lines of (mainly systemic) treatment. Here, we present a dosimetric comparison of three different radiotherapy modalities (HDR, SBRT, eBT) with an equivalent prescription dose of 25 Gy in the context of solitary (technically or functionally) unresectable liver metastasis, to determine differences in target volume coverage and OAR dose distribution. Overall, SBRT had superior target volume coverage and the fastest delivery time, however, it also resulted in significantly higher doses to the surrounding healthy tissues, potentially resulting in relevant radiation-induced toxicity. On the other hand, SBRT poses no increased risks of intervention-related complications (e.g. bleeding, cholestasis) in comparison with invasive HDR and eBT. Apart from these theoretical dosimetric properties and differences, other practical and logistical considerations are to be made upon selecting the appropriate radiation technique for an individual clinical patient scenario. Pros and cons are discussed in Table 2.

**Table 2:** Pros and cons of different radiotherapy modalities in the context of unresectable solitary unresectable liver metastasis. HDR = high dose-rate brachytherapy; SBRT = stereotactic body radiation therapy; eBT = electronic brachytherapy.

	HDR	SBRT	eBT
Invasiveness	minimally invasive	non-invasive^a^	minimally invasive
Seeding	possible	impossible	possible
Required expertise	high	medium	high
Margin	no	required	no
Fractionation	discouraged	possible	discouraged
Dose escalation in target	always	possible	always

a Implantation of fiducials is possible.

One of the main reasons for the higher OAR dose exposure in SBRT plans is the mandatory use of an isotropic expansion around the GTV to correct for setup uncertainties, e.g. due to breathing. Deep inspiration breath-hold and respiratory gating have already reduced these margins, resulting in better OAR sparing and potentially allowing further dose-escalation up to 35 − 40 Gy[21]. Further refinement of SBRT treatment and setup techniques, e.g. the use of online adaptive magnetic resonance-guidance, results in superior target coverage and improved OAR sparing, however, at the expense of increased treatment time and costs [4,23].

Electronic BT demonstrated excellent OAR dose sparing, yet showed shortcomings regarding target coverage. In individual cases, the extent of these questions its applicability in a clinical setting. Low-energy X-rays are emitted from the tip of the applicator, resulting in a spherical dose delivery with a steep gradient, yielding excellent homogeneity for spherical target volumes. Liver malignancies, however, usually exhibit more diffuse growth patterns, resulting in irregular, asymmetrical shapes. Overshooting these boundaries results in unnecessary healthy liver exposure, while undershooting poses the risk of undertreatment. A potential way to overcome this might be the development of a multi-dwelling approach, i.e. through multicatheter implantation, as in HDR. Previously investigated for locally inoperable tumors, this allows for a very conformal treatment with superior D90% in comparison with permanent seed implantations, while also requiring fewer trajectories to achieve this [24]. Furthermore, the possibility of non-coplanar multicatheter implantation as in HDR enables anatomy-oriented three-dimensional dose planning and optimization. This allows treatment with adequate coverage of irregularly shaped tumours, regardless of size (this in contrast to SBRT), while still keeping the OAR exposure to a minimum. This does, however, require further investigation. The combination of eBT with surgical robotic systems is also being investigated [25,26]. The assessment of the equivalent uniform dose (EUD) would also be of interest, as this may show an additional advantage of HDR/eBT since the dose delivered at the center of the lesion will be much higher in comparison with a homogeneous SBRT plan as outlined in Table 2. Furthermore, the use of eBT for solid tumor lesions (in comparison with a resection cavity) requires further analysis. Dosimetry and Monte Carlo modelling using 50 kV beams have been validated in the context of contact brachytherapy for macroscopic rectal tumors [27].

In patients with primary liver malignancies (e.g. hepatocellular carcinoma [HCC]), sparing healthy liver tissue is of particular importance, as these tumors commonly develop on a background of cirrhosis due to chronic alcohol abuse or hepatitis, implicating limited baseline liver function. Radiotherapy has been shown to improve survival time in patients with unresectable HCC [28]. In a comparative analysis between HDR and SBRT, Hass *et al.* compared 85 patients who were treated with HDR (15 − 20 Gy, depending on histology) [11]. Healthy liver exposure was significantly reduced for HDR in the 20-Gy group. In another series of 38 patients with unresectable HCC by Walter *et al.*, healthy liver sparing was again superior for HDR in comparison with SBRT for the same lesions, while maintaining excellent target conformity [29]. However, the prescribed dose differed between groups (1 × 15 Gy versus 3 × 12.5 Gy). Finally, in a dosimetric comparison by Wust *et al.* (20 Gy in a single fraction), HDR showed excellent properties when compared to different linac-based radiotherapy modalities, especially for lesions < 3 cm [30]. Clinical data suggest that SBRT is a well-tolerated local treatment modality yielding excellent local control with acceptable toxicity and preservation of quality of life in HCC patients [31].

Electronic BT (20 − 30 Gy; in this context more commonly referred to as IORT) has become an established adjuvant treatment option for patients with resectable brain metastases, with comparable long-term outcome in terms of local control and overall survival compared to SBRT of the resection cavity[32,33]. Furthermore, risk of radiation necrosis is low and the fast-track approach (i.e. one-stop treatment) results in faster completion of interdisciplinary local treatment and thus shorter time to next treatment[34,35]. The translation of these properties to the context of liver metastases requires further attention in prospective trials.

This trial carries certain limitations. Its retrospective nature and relatively small sample size prevent generalizability of the results to every clinical scenario. A dose of 25 Gy was chosen to allow head-to-head comparison of the different endpoints, its biological efficacy should, however, be established in prospective trials. Future trials should also investigate the time it takes to develop treatment plans across all modalities and investigate differences for patients with multiple liver metastases. To the best of our knowledge, we present the first direct dosimetric comparison for non-resected liver metastases including an eBT arm as a potential future alternative for liver tissue-preserving metastasis-directed therapy.

## Conclusion

The role of definitive radiotherapy for unresectable liver metastases is emerging rapidly. Minimally invasive eBT with a single needle applicator has the potential to deliver local irradiation while limiting dose to healthy liver tissue and neighboring OARs in comparison with HDR and SBRT. Here, we highlighted *in silico* feasibility for spherical metastases treated with a single catheter. A multicatheter approach (with anatomy-oriented dose optimization) as in HDR might overcome current limitations, optimizing target volume coverage while maintaining the strengths of kilovoltage irradiation. Future prospective investigations are required to define the role of eBT in the spectrum of liver-directed therapies.

## CRediT authorship contribution statement

**Cas Stefaan Dejonckheere:** Data curation, Investigation, Methodology, Project administration, Validation, Visualization, Writing – original draft, Writing – review & editing. **Mateusz Bilski:** Conceptualization, Investigation, Methodology, Supervision, Validation, Writing – review & editing. **Younèss Nour:** Writing – review & editing. **Davide Scafa:** Writing – review & editing. **Paweł Cisek:** Investigation, Writing – review & editing. **Katarzyna Korab:** Investigation, Writing – review & editing. **Julia Ponikowska:** Investigation, Writing – review & editing. **Ewa Wojtyna:** Investigation, Writing – review & editing. **Sylwia Sroka:** Investigation, Writing – review & editing. **Fabian Kugel:** Investigation, Writing – review & editing. **Molina Grimmer:** Writing – review & editing. **Jasmin Holz:** Writing – review & editing. **Stephan Garbe:** Writing – review & editing. **Patrick Eich:** Writing – review & editing. **Eleni Gkika:** Writing – review & editing. **Gustavo Renato Sarria:** Conceptualization, Investigation, Methodology, Supervision, Validation, Writing – review & editing. **Julian Philipp Layer:** Data curation, Formal analysis, Investigation, Methodology, Validation, Visualization, Writing – review & editing.

## Funding

The authors declare that no funds, grants, or other support were received during the preparation of this manuscript.

## Declaration of competing interest

The authors declare the following financial interests/personal relationships which may be considered as potential competing interests: JPL reports travel expenses from Carl Zeiss Meditec not related to this work; stocks and travel expenses from TME Pharma, stocks and honoraria from Siemens Healthineers and stocks from Bayer and BioNTech, all unrelated to this work. GRS reports travel expenses and personal fees from Carl Zeiss Meditec. Speaker bureau and advisory board at Carl Zeiss Meditec. Personal fees from MedWave Clinical Trials. Personal fees from AstraZeneca. Personal fees and travel expenses from Guerbet. None of the above are related to this work. All remaining others have no conflict of interest to disclose.

## Data Availability

Individual patient data can be made available upon reasonable request to the corresponding author.
