# Supplementary material for: A dosimetric comparison of different radiotherapy modalities for Non-Resected oligometastatic liver Disease
Source: Clin Transl Radiat Oncol. 2025 Mar 13;52:100947. doi: 10.1016/j.ctro.2025.100947 (PMC11937280; doi:10.1016/j.ctro.2025.100947)
Supplement: Supplementary Data 1 [file mmc1.docx]

**SUPPLEMENT**

***Supplementary Table 1***. Summary of all lesion characteristics and dosimetric data (*n* = 30). eBT = electronic brachytherapy; HDR = high dose-rate brachytherapy; SBRT = stereotactic body radiation therapy; GTV = gross tumor volume; CTV = clinical target volume; PTV = planning target volume; Gy = Gray; HI = homogeneity index.

| **Parameter** | **Median (range)** | ***p*-value** |
| --- | --- | --- |
| Lesion diameter (mm) | 26.7 (15.2–38.2) |  |
| Lesion volume (cm^3^) | 9.3 (2.5–29.7) |  |
| Uninvolved liver volume (cm^3^) | 1450.2 (1085.8–2227.2) |  |
| Target volume (cm^3^) |  |  |
| eBT (GTV) | 9.3 (2.5–29.7) | > 0.99 |
| HDR (GTV) | 9.3 (2.5–29.7) | < 0.001 |
| SBRT (PTV) | 25.6 (11.4–59.2) | < 0.001 |
| CTV D98% (Gy) |  |  |
| eBT | 11.4 (3.5–16.5) | < 0.001 |
| HDR | 23.1 (19.8–25.7) | < 0.001 |
| SBRT | 29.2 (23.2–33.1) | < 0.001 |
| CTV D95% (Gy) |  |  |
| eBT | 14.9 (4.7–21.9) | < 0.001 |
| HDR | 25.3 (22.0–27.9) | < 0.001 |
| SBRT | 29.5 (25.4–33.5) | < 0.001 |
| CTV D95% (%) |  |  |
| eBT | 59.6 (18.8–87.6) | < 0.001 |
| HDR | 101.4 (88.2–111.5) | < 0.001 |
| SBRT | 117.8 (101.6–133.8) | < 0.001 |
| CTV D90% (Gy) |  |  |
| eBT | 19.1 (6.7–27.5) | < 0.001 |
| HDR | 28.4 (25.4–30.9) | < 0.001 |
| SBRT | 29.7 (27.9–33.8) | < 0.001 |
| CTV D90% (%) |  |  |
| eBT | 76.4 (26.8–110.0) | < 0.001 |
| HDR | 113.5 (101.6–123.6) | < 0.001 |
| SBRT | 118.9 (111.8–135.2) | < 0.001 |
| CTV D80% (Gy) |  |  |
| eBT | 24.7 (11.1–36.1) | < 0.001 |
| HDR | 33.9 (30.4–38) | < 0.001 |
| SBRT | 30.1 (29.7–32.3) | < 0.001 |
| CTV D80% (%) |  |  |
| eBT | 98.8 (44.4–144.4) | < 0.001 |
| HDR | 135.4 (121.6–151.8) | < 0.001 |
| SBRT | 120.6 (118.8–129.0) | < 0.001 |
| CTV D50% (Gy) |  |  |
| eBT | 44.4 (24.9–69.9) | 0.04 |
| HDR | 48.7 (40.7–64.0) | < 0.001 |
| SBRT | 31.1 (30.5–35.2) | < 0.001 |
| HI |  |  |
| eBT | –0.2 (–0.4 to –0.1) | < 0.001 |
| HDR | 3.6 (2.2–6.5) | < 0.001 |
| SBRT | 0.3 (0.2–0.7) | < 0.001 |
| Uninvolved liver V9.1Gy (cm^3^) |  |  |
| eBT | 13.8 (3.4–41.6) | < 0.001 |
| HDR | 49.2 (12.7–116.8) | < 0.001 |
| SBRT | 98.8 (54.3–303.7) | < 0.001 |
| Uninvolved liver D66% (Gy) |  |  |
| eBT | 0.1 (0.1–0.5) | < 0.001 |
| HDR | 0.6 (0.2–2.0) | < 0.001 |
| SBRT | 0.3 (0.1–1.2) | 0.005 |
| Spinal canal Dmax (Gy) |  |  |
| eBT | 0.0 (0.0–0.9) | < 0.001 |
| HDR | 0.4 (0.1–2.7) | < 0.001 |
| SBRT | 2.1 (0.8–6.7) | < 0.001 |
| Spinal canal D0.035 (Gy) |  |  |
| eBT | 0.0 (0.0–0.7) | < 0.001 |
| HDR | 0.4 (0.1–2.4) | < 0.001 |
| SBRT | 1.9 (0.8–20.7) | < 0.001 |
| Spinal canal V7Gy (cm^3^) |  |  |
| eBT | 0.0 (0.0–0.0) | > 0.99 |
| HDR | 0.0 (0.0–0.0) | > 0.99 |
| SBRT | 0.0 (0.0–0.0) | > 0.99 |
| Spinal canal D1cm^3^ (Gy) |  |  |
| eBT | 0.0 (0.0–0.3) | < 0.001 |
| HDR | 0.4 (0.1–67.0) | < 0.001 |
| SBRT | 1.8 (0.7–5.8) | < 0.001 |
| Stomach Dmax (Gy) |  |  |
| eBT | 0.0 (0.0–6.3) | < 0.001 |
| HDR | 0.7 (0.2–8.0) | < 0.001 |
| SBRT | 2.0 (0.4–11.9) | < 0.001 |
| Stomach V11.2Gy (cm^3^) |  |  |
| eBT | 0.0 (0.0–0.0) | > 0.99 |
| HDR | 0.0 (0.0–0.0) | > 0.99 |
| SBRT | 0.0 (0.0–0.1) | > 0.99 |
| Stomach D1cm^3^ (Gy) |  |  |
| eBT | 0.0 (0.0–3.7) | < 0.001 |
| HDR | 0.6 (0.2–5.6) | < 0.001 |
| SBRT | 1.8 (0.4–9.1) | < 0.001 |
| Stomach V17.4Gy (cm^3^) |  |  |
| eBT | 0.0 (0.0–0.0) | > 0.99 |
| HDR | 0.0 (0.0–0.0) | > 0.99 |
| SBRT | 0.0 (0.0–0.0) | > 0.99 |
| Stomach D5cm^3^ (Gy) |  |  |
| eBT | 0.0 (0.0–0.0) | < 0.001 |
| HDR | 0.5 (0.2–3.5) | < 0.001 |
| SBRT | 1.5 (0.2–7.2) | < 0.001 |
| Biliary tract Dmax (Gy) |  |  |
| eBT | 0.0 (0.0–8.1) | < 0.001 |
| HDR | 1.7 (0.5–20.8) | 0.07 |
| SBRT | 2.7 (0.1–22.0) | < 0.001 |
| Duodenum Dmax (Gy |  |  |
| eBT | 0.0 (0.0–5.3) | < 0.001 |
| HDR | 1.2 (0.1–6.7) | 0.58 |
| SBRT | 0.4 (0.1–11.2) | < 0.001 |
| Duodenum V11.2Gy (cm^3^) |  |  |
| eBT | 0.0 (0.0–0.3) | > 0.99 |
| HDR | 0.0 (0.0–0.0) | > 0.99 |
| SBRT | 0.0 (0.0–0.0) | > 0.99 |
| Duodenum V9 Gy (cm^3^) |  |  |
| eBT | 0.0 (0.0–0.0) | > 0.99 |
| HDR | 0.0 (0.0–0.0) | > 0.99 |
| SBRT | 0.0 (0.0–0.0) | > 0.99 |
| Duodenum D1cm^3^ (Gy) |  |  |
| eBT | 0.0 (0.0–3.3) | < 0.001 |
| HDR | 1.0 (0.1–4.9) | 0.30 |
| SBRT | 0.3 (0.0–7.8) | < 0.001 |
| Right kidney D1cm^3^ (Gy) |  |  |
| eBT | 0.0 (0.0–7.7) | < 0.001 |
| HDR | 1.3 (0.0–16.3) | 0.37 |
| SBRT | 0.4 (0.0–16.4) | < 0.001 |
| Right kidney V10 Gy (%) |  |  |
| eBT | 0.0 (0.0–0.6) | 0.50 |
| HDR | 0.0 (0.0–3.4) | 0.50 |
| SBRT | 0.0 (0.0–2.7) | 0.25 |
| Heart Dmax (Gy |  |  |
| eBT | 0.0 (0.0–2.1) | < 0.001 |
| HDR | 0.6 (0.2–5.6) | 0.24 |
| SBRT | 0.9 (0.1–7.3) | < 0.001 |
| Rib D1cm^3^ (Gy) |  |  |
| eBT | 7.2 (0.0–105.9) | 0.10 |
| HDR | 8.1 (1.1–48.5) | < 0.001 |
| SBRT | 16.6 (8.0–29.3) | < 0.001 |
| Great vessels D1cm^3^ (Gy) |  |  |
| eBT | 0.0 (0.0–0.1) | < 0.001 |
| HDR | 0.6 (0.2–2.2) | < 0.001 |
| SBRT | 2.6 (1.0–5.3) | < 0.001 |
| Gallbladder Dmax (Gy) |  |  |
| eBT | 0.0 (0.0–61.7) | < 0.001 |
| HDR | 2.2 (0.7–34.5) | 0.35 |
| SBRT | 2.0 (0.2–20.3) | 0.005 |
| Bowel Dmax (Gy) |  |  |
| eBT | 0.0 (0.0–29.1) | < 0.001 |
| HDR | 1.2 (0.3–18.2) | 0.65 |
| SBRT | 1.6 (0.2–15.4) | < 0.001 |
| Bowel V11.9Gy (cm^3^) |  |  |
| eBT | 0.0 (0.0–1.6) | > 0.99 |
| HDR | 0.0 (0.0–2.5) | 0.50 |
| SBRT | 0.0 (0.0–3.0) | 0.50 |
| Bowel D5cm^3^ (Gy) |  |  |
| eBT |  |  |
| HDR | 1.0 (0.3–10.2) | > 0.99 |
| SBRT | 1.1 (0.1–10.8) |  |
| Esophagus Dmax (Gy) |  |  |
| eBT | 0.0 (0.0–1.3) | < 0.001 |
| HDR | 0.5 (0.2–4.3) | < 0.001 |
| SBRT | 1.6 (0.0–8.0) | < 0.001 |
| Esophagus D1cm^3^ (Gy) |  |  |
| eBT | 0.0 (0.0–0.7) | < 0.001 |
| HDR | 0.5 (0.1–3.4) | < 0.001 |
| SBRT | 1.3 (0.1–6.7) | < 0.001 |
